# Genomic Analysis of Bovine Staphylococcus aureus Isolates from Milk To Elucidate Diversity and Determine the Distributions of Antimicrobial and Virulence Genes and Their Association with Mastitis

**DOI:** 10.1128/mSystems.00063-20

**Published:** 2020-07-07

**Authors:** Sohail Naushad, Diego B. Nobrega, S. Ali Naqvi, Herman W. Barkema, Jeroen De Buck

**Affiliations:** a Department of Production Animal Health, Faculty of Veterinary Medicine, University of Calgary, Calgary, Alberta, Canada; b Canadian Bovine Mastitis and Milk Quality Research Network, Saint-Hyacinthe, Quebec, Canada; University of Illinois at Urbana-Champaign

**Keywords:** intramammary infection, *Staphylococcus aureus*, whole-genome sequencing, virulence factors, antimicrobial resistance genes, sequence types, clonal complex, Spa types, AMR, adherence, intramammary infection, mastitis

## Abstract

Staphylococcus aureus causes persistent clinical and subclinical bovine intramammary infections (IMI) worldwide. However, there is a lack of comprehensive information regarding genetic diversity, the presence of antimicrobial resistance (AMR), and virulence genes for S. aureus in bovine milk in Canada. Here, we performed whole-genome sequencing (WGS) of 119 Canadian bovine milk S. aureus isolates and determined they belonged to 8 sequence types (ST151, ST352, ST351, ST2187, ST2270, ST126, ST133, and ST8), 5 clonal complexes (CC151, CC97, CC126, CC133, and CC8), and 18 distinct Spa types. Pan-, core, and accessory genomes were composed of 6,340, 1,279, and 2,431 genes, respectively. Based on phenotypic screening for AMR, resistance was common against beta-lactams (19% of isolates) and sulfonamides (7% of isolates), whereas resistance against pirlimycin, tetracycline, ceftiofur, and erythromycin and to the combination of penicillin and novobiocin was uncommon (3, 3, 3, 2, and 2% of all isolates, respectively). We also determined distributions of 191 virulence factors (VFs) in 119 S. aureus isolates after classifying them into 5 functional categories (adherence [*n* = 28], exoenzymes [*n* = 21], immune evasion [*n* = 20], iron metabolism [*n* = 29], and toxins [*n* = 93]). Additionally, we calculated the pathogenic potential of distinct CCs and STs and determined that CC151 (ST151 and ST351) had the highest pathogenic potential (calculated by subtracting core-VFs from total VFs), followed by CC97 (ST352 and ST2187) and CC126 (ST126 and ST2270), potentially linked to their higher prevalence in bovine IMI worldwide. However, there was no statistically significant link between the presence of VF genes and mastitis.

**IMPORTANCE** Staphylococcus aureus is a major cause of bovine intramammary infections, leading to significant economic losses to dairy industry in Canada and worldwide. There is a lack of knowledge regarding genetic diversity, the presence of antimicrobial resistance (AMR), and virulence genes for S. aureus isolated from bovine milk in Canada. Based on whole-genome sequencing and genomic analysis, we have determined the phylogeny and diversity of S. aureus in bovine milk and concluded that it had a large accessory genome, limited distribution of AMR genes, variable VF gene profiles and sequence types (ST), and clonal complex (CC)-specific pathogenic potentials. Comprehensive information on the population structure, as well as the virulence and resistance characteristics of S. aureus from bovine milk, will allow for source attribution, risk assessment, and improved therapeutic approaches in cattle.

## Abstract (precis)

Staphylococcus aureus is a major cause of bovine intramammary infections, leading to significant economic losses to dairy industry in Canada and worldwide. There is a lack of knowledge regarding genetic diversity, the presence of antimicrobial resistance (AMR), and virulence genes for S. aureus isolated from bovine milk in Canada. Based on whole-genome sequencing and genomic analysis, we have determined the phylogeny and diversity of S. aureus in bovine milk and concluded that it had a large accessory genome, limited distribution of AMR genes, variable VF gene profiles and sequence types (ST), and clonal complex (CC)-specific pathogenic potentials. Comprehensive information on the population structure, as well as the virulence and resistance characteristics of S. aureus from bovine milk, will allow for source attribution, risk assessment, and improved therapeutic approaches in cattle.

## INTRODUCTION

Staphylococcus aureus can cause acute and chronic infections associated with high morbidity in a wide variety of hosts, including humans and farmed and companion animals (1–3). In the dairy industry, S. aureus causes persistent clinical and subclinical intramammary infections (IMI) (4). Staphylococcus aureus IMIs, often spread during the milking process, can result in chronic infections, often persisting for the life of the animal (4–6), causing tissue damage, reduced milk quality and production, and increased individual cow and bulk tank milk somatic cell count (SCC) (4, 7, 8). Staphylococcus aureus IMI reduces animal health and welfare and poses a biosafety hazard for raw dairy products, leading to substantial economic losses in the dairy industry worldwide (4, 9). The pathogenesis of S. aureus mastitis is complex: it starts with colonization of the teat end and infection subsequently spreading into the intramammary space, either by progressive colonization or changes in intramammary pressure caused by the milking machines (4, 10). In the mammary alveolus, S. aureus attaches to and internalizes into mammary epithelial cells, where it multiplies and establishes a chronic IMI (10–12). Mechanisms by which S. aureus IMI is established and maintained in dairy cows are not fully understood but generally involve both host immune escape and modulation strategies (13, 14). In addition, a number of studies have suggested biofilm formation as a mechanism by which S. aureus establishes and maintains itself in dairy cows (15–17). Genetic regulatory circuits that control S. aureus adaptation and virulence are complex, involving signals from the external environment to modulate the production of a wide arsenal of cell surface and extracellular proteins, known as virulence factors (VFs) (13, 14, 18).

Extensive use of antimicrobials to treat bovine mastitis and for dry cow therapy exerts selective pressure on S. aureus, leading to the emergence and spread of antimicrobial-resistant (AMR) S. aureus strains (19–22). In particular, the emergence of multiple-drug-resistant (MDR) strains can become a major challenge in the treatment of bovine mastitis and is a growing concern for public health (23–26), as methicillin-resistant S. aureus (MRSA) has been reported in human and veterinary medicine (25).

During the past decade, the molecular epidemiology of S. aureus IMI in dairy cattle has been studied using various methods, including electrophoretic comparison techniques, such as pulsed-field gel electrophoresis (PFGE) (27), random amplification of polymorphic DNA (RAPD) analysis (28), multilocus enzyme electrophoresis (MLEE) (29), and sequence-based typing schemes, such as multiple-locus sequence typing (MLST) (30), staphylococcal protein A (spa) typing (31), and multiple-locus VNTR (variable number of tandem repeats) analysis (MLVA) (32). Although these methods were helpful in typing S. aureus strains, they failed to reveal fine details of genetic differences between strains. However, whole-genome sequencing (WGS) of bacterial genomes has become the preferred method to understand microevolution, phylogenies, and inter- and intraspecies differences (33–35). By providing definitive genotype information, WGS offers the highest practical resolution for matching strain diversity with resistance and virulence determinants (36–38).

Objectives of this study were to use the WGS data of 119 bovine S. aureus isolates to elucidate (i) molecular types (MLST and spa types), (ii) genetic diversity and evolutionary relationships, by constructing pan- and core genomes and phylogenetic trees based on multilocus sequence analysis (MLSA) and single-nucleotide polymorphisms (SNPs), (iii) AMR gene (ARGs) profiles, (iv) distributions of VFs, and (v) associations among genotypes, ARGs, and VF-derived potential to cause mastitis.

## RESULTS

### Identification and distribution of STs and spa types.

**FIG 1:**
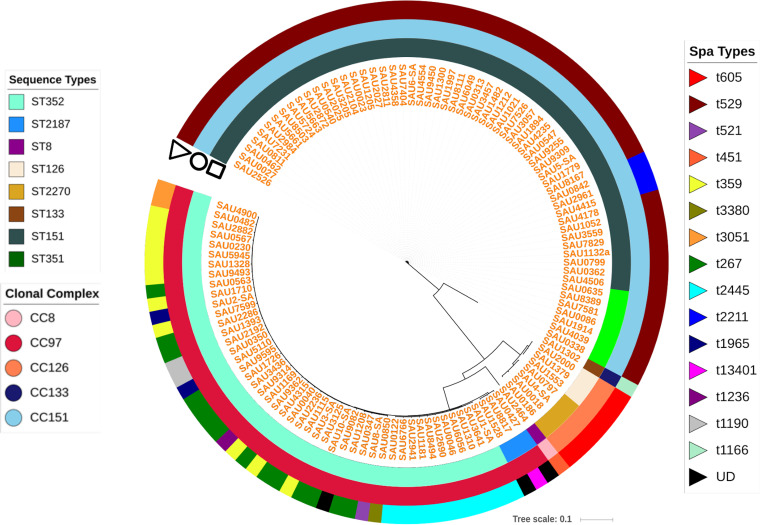
Core SNP-based phylogenetic tree of distribution of sequence types (STs), clonal complexes (CCs), and spa types. The SNP tree indicates phylogenetic relationships among 119 Staphylococcus aureus isolates recovered from bovine milk. This tree was constructed with Parsnp v1.2 (123) and was overlayed with information regarding STs, CCs, and spa types using iTOL v4 (147). The first ring indicates the distribution of 119 isolates into 8 distinct STs (ST151, ST352, ST351, ST2187, ST2270, ST126, ST133, and ST8). The second (middle) ring indicates grouping of STs into 5 CCs (CC151, CC97, CC126, CC133, and CC8), whereas the third (outer) ring indicates distribution of spa types. For 21 isolates, a spa type reference was not available in the reference database; hence, spa types for those could not be determined. These isolates were labeled UD (undetermined) in the outer circle.

MLST analysis grouped 119 S. aureus isolates (Table 1) into 8 STs and 5 distinct CCs (Fig. 1). The majority of isolates were assigned to ST151 (*n* = 54 [45%]), followed by ST352 (*n* = 46 [39%]). For the remaining isolates, ST351, ST2187, ST2270, and ST126 had 7, 3, 4, and 3 isolates, respectively, whereas ST133 and ST8 were singletons (Fig. 1). The eBURST analysis of STs clustered ST151 and ST351 into CC151 (51% isolates), ST2187 and ST352 into CC97 (41% isolates), and ST2270 and ST126 into CC126 (6.4%), whereas ST133 and ST8 isolates were part of CC133 and CC8, respectively (Fig. 1). Spa typing identified 119 S. aureus isolates into 18 distinct spa types (Fig. 1).

**TABLE 1:** Number of isolates and unique herds grouped by SCC level and region of origin

Origin	No. of isolates (no. of unique herds) from randomly selected, nonclinical cows				
	Low SCC (≤150,00 cells/ml)	Medium SCC (150,00–250,000 cells/ml)	High SCC (>250,000 cells/ml)	Clinical mastitis	Total
Alberta	1 (1)	0	5 (4)	5 (4)	11 (6)
Ontario	3 (3)	1 (1)	16 (10)	18 (12)	38 (19)
Quebec	2 (2)	0	20 (9)	22 (10)	44 (15)
Atlantic Canada	5 (4)	0	2 (2)	19 (9)	26 (10)
Total	11 (10)	1 (1)	43 (25)	64 (35)	119 (50)

### Phylogenetic analyses.

The majority of interrelationships at major clades were similar and fairly consistent among phylogenetic trees constructed using a core set of proteins, PhyloPhlAn, core-SNPs, and MLSA. However, many relationships were observed toward the tip of the trees (data not shown). All trees had branching of S. aureus isolates into 8 nodes and 5 main clades that corresponded well with STs and CCs predicted by eBURST analysis (Fig. 1).

### Pan-genome analysis.

**FIG 2:**
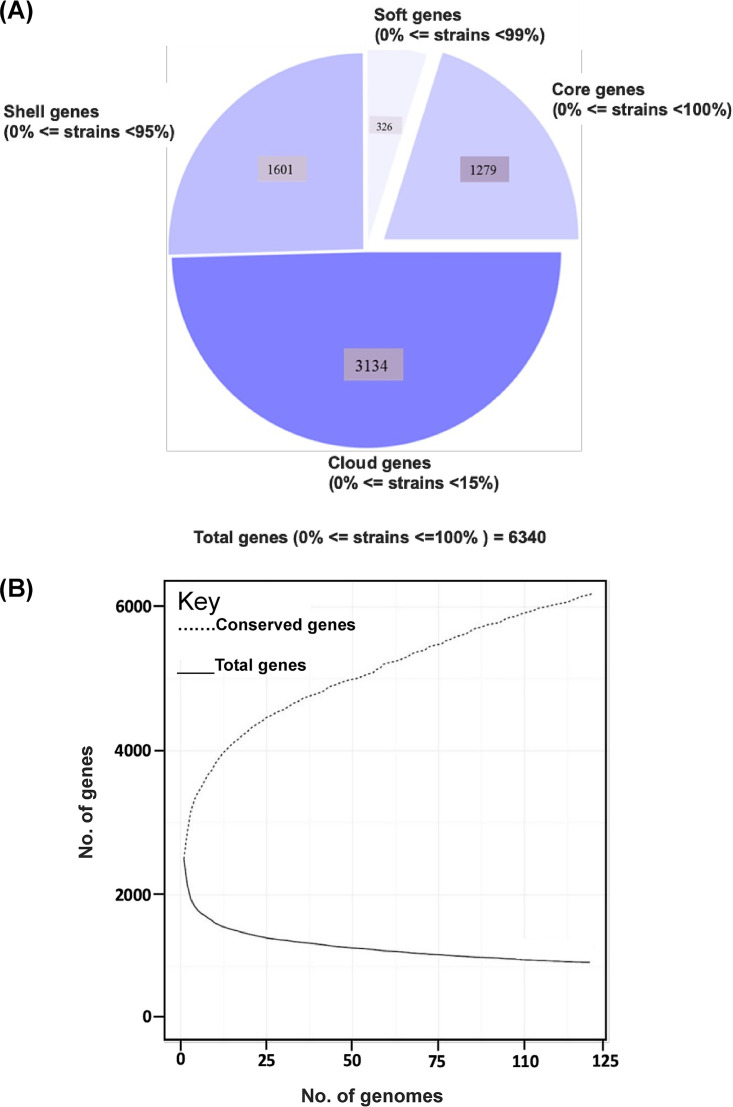
Pan-genome of 119 S. aureus isolates. (A) Distribution of pan-genome into core, soft-core, shell, and cloud categories. (B) Changes in the total number of genes versus conserved genes upon addition of each individual S. aureus genome.

**FIG 3:**
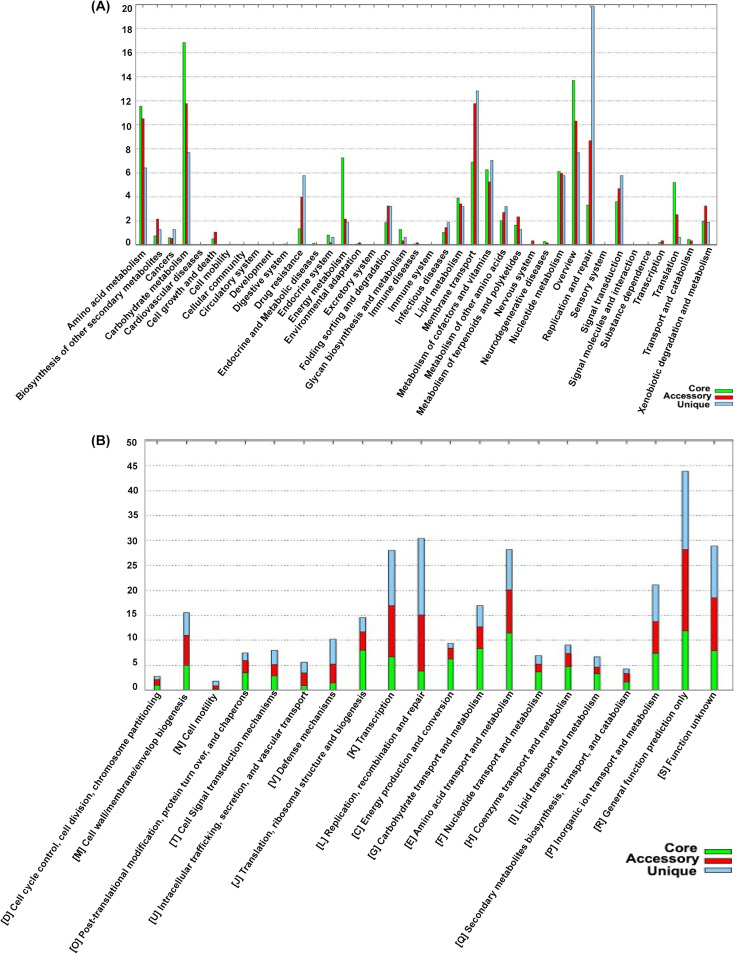
Pan-genome characterization based on distribution of functional categories. Distribution of pan-genome into functional categories obtained after comparing the pan-genome with KEGG (A) and COG (B) reference databases.

The pan-genome of 119 S. aureus isolates tested in this study had 6,340 genes. The core genome (shared by >99% of S. aureus isolates) consisted of 1,279 genes. The accessory genome (genes in >2 isolates but not in all) consisted of 2,431 genes, and the unique genome was composed of 2,845 genes. The soft core, shell, and cloud contained 326, 1,601, and 3,134 genes, respectively (Fig. 2). Based on a rarefaction curve after inclusion of ∼90 (75%) isolates into the analyses, the number of core genes remained fairly constant at ∼1,300 genes, whereas the total number of genes in the pan-genome continued to increase (Fig. 2). Functional annotation of genes in the pan-genome performed using the COG and KEGG databases revealed a distribution of functional categories among 3 pan-genome sets (Fig. 3). The largest fraction of the core genome consisted of genes involved in housekeeping processes, include transcription, translation, ribosomal structure and biogenesis, and RNA processing and modification, whereas a smaller fraction of housekeeping gene content was in the accessory and unique genomes.

### Distributions and associations of virulence genes.

Among the 28 adherence-related genes, 12 genes (*alt*, *clfA*, *clfB*, *ebp*, *ebh*, *efb*, *fnbA*, *eap-map*, *icaA*, *icaB*, *icaC*, and *icaD*) were present in ≥90% of S. aureus isolates (Table 2). Seven adherence genes (*aap*, *cna*, *uafA*, *sdrF*, *sdrG*, *sdrH*, and *sdrI*) were absent from all isolates. The biofilm-associated gene, *bap*, was detected in only 2 isolates from ST2270. Some of these 28 genes had ST-specific distributions. For example, *fnbB* was detected in ST8, ST133, ST352, and ST2270, *sasC* was detected in all STs except CC151 (ST151 and ST351), and *sasG* was detected only in CC5 (ST352 and ST2187). The *sraP* was detected in ST8, ST133, ST352, ST2187, and ST126, whereas *sdrC* was detected in ST133, ST352, ST2187, and ST2270 and *sdrD* was detected in ST8, ST133, and ST352 (Table 2).

**TABLE 2:**
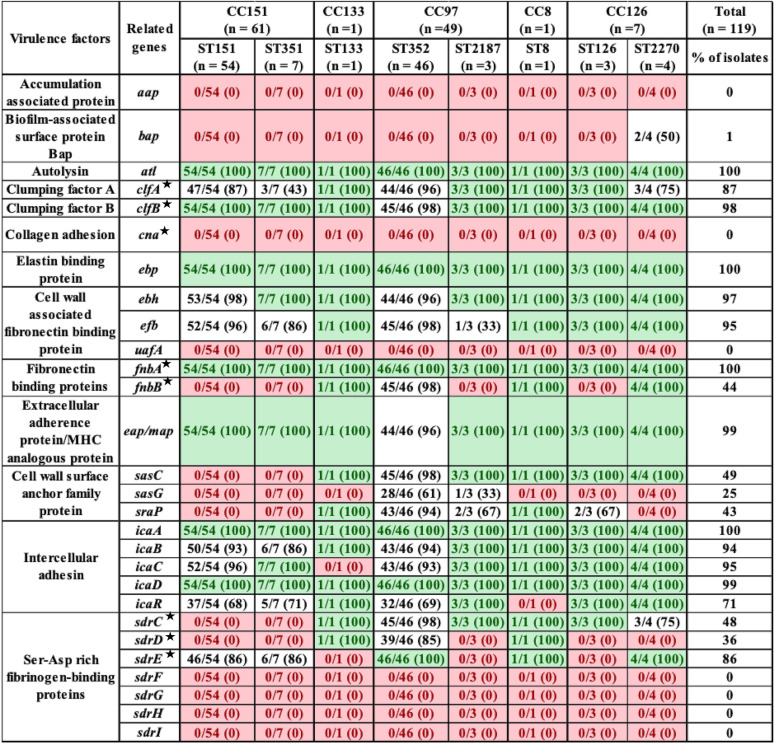
Distribution of adherence related virulence factors in S. aureus isolates from bovine milk, grouped according to ST and CC^*a*^

a A star indicates genes that encode microbial surface component recognizing adhesive matrix molecules (MSCRAMMs). Light green indicates gene was in all isolates within a given ST; red indicates a gene that was not identified in any isolates within a given ST. Values in parentheses indicate the percentage of isolates that contained a particular VF.

With respect to the 21 exoenzymes, *sspD*, *sspE*, *sspF*, and *sak* were not detected in any isolates, but *adsA*, *aur*, *sspA*, *sspB*, *sspC*, *hysA*, *lip*, *geh*, *splA*, *splB*, *splC*, *splD*, *splE*, *splF*, *coa*, and *vWbp* were detected in 90 to 100% of isolates. In contrast, *vWbp* was detected in ST8 only (represented by 1 isolate) (Table 3). Exoenzymes *geh* and *splA* were not detected in ST133. Similarly, *splE* was detected in ST2187 and ST133, and *coa* was not present in ST133 (Table 3). With respect to the 20 host immune evasion genes, capsular genes (*capA* to *capP*) were detected in all STs, except *capH* and *capK* were not detected in ST133 and *capJ* and *capI* were not detected in ST8 and ST2270, respectively (Table 4). Chemotaxis inhibitory protein (*chp*) and staphylococcal complement inhibitor (*scn*) was not detected in any isolate. Staphylococcal protein A (*spa*) and *sbi* were detected in 100% and 94% of isolates (Table 4). The identification and distribution of genes related to iron uptake and metabolism were uniform among S. aureus isolates, with all 29 genes detected in almost all isolates, except *isdB* and *srtB* were not detected in ST126 and ST8, respectively (Table 5). Type VII secretion system genes (*esaA*, *esaB*, *esaC*, *essA*, *essB*, *essC*, *esxA*, and *esxB*) were detected in most isolates, although *esaC* and *essA* were exclusively absent from CC126 and CC151 and *essC* and *esxA* were not detected in CC8 (Table 6). With respect to phenol-soluble modulins (PSM), except for *PSMβ1* and *PSMβ2*, which were detected in 99% and 96% of all S. aureus isolates, other *PSMβ* genes (*PSMβ3*, *PSMβ4*, *PSMβ5*, and *PSMβ6*) and *PSMα* genes (*PSMα1*, *PSMα2*, *PSMα3*, *PSMα4*, and *PSMmec*) were not detected (Table 6). Among hemolysins, alpha (*hla*), beta (*hlb*), and gamma (*hlgA*, *hlgB*, and *hlgC*) hemolysins were detected in almost all isolates, except for *hla* and *hlgA*, which were not detected in ST8 and ST126, respectively (Table 6), and *hld* was detected in CC5 (ST352 and ST2187) and CC126 (ST126 and ST2187). Among leukocidins, *lukF-*like was detected in all isolates, whereas *lukM* was only present in CC151 and ST352. Although *lukF-PV* was in all S. aureus isolates, *lukS-PV* was detected only in ST1351 (29%), ST352 (9%), and ST2270 (75%). Concerning leukotoxins, both *lukD* and *lukE* were detected in 99% of isolates. The toxic shock syndrome toxin (*tsst*) gene was detected in ST351 (7/7) and ST151 (1/54) only (Table 6). Concerning exfoliative toxins, *eta* was identified in all isolates except for ST133, whereas *etb*, *etc*, and *etd* were not detected (Table 6). Enterotoxins (*n* = 21) were not detected in most of our 119 isolates, except in ST151 and ST351, which contained (*sed*, *seg*, *sei*, *sell*, *selm*, *seln*, *selo*, *selu*, *selv*, *yent1*, and *yent2*) (Table 7). Most exotoxin (*set*) genes (*n* = 34) were uniformly distributed and present in ≥90% of isolates (Table 7), except *set2*, *set15*, *set21*, *set30*, *set35*, and *set40*. One of the *set* genes (*set26*) had clone-specific distribution and was detected in 100% of isolates of CC151 (ST151 and ST351). Apart from establishing the distribution of VFs among 119 S. aureus isolates, the total number of genes unique to each ST was calculated. On average, 127 VF genes were detected in all STs, with the highest number of genes (135 VFs) detected in ST151 and ST352, followed by ST351 and ST2187, which contained 134 and 129 VF genes, respectively. Slightly fewer genes were detected in ST2270 (*n* = 127) and ST8 (*n* = 126). The fewest genes were in ST133 (*n* = 121) and ST126 (*n* = 120). A large number (83/191) of VFs detected in all STs were defined as core VFs. The pathogenic potential of various STs and CCs, calculated by subtracting core VFs (commonly detected among all STs or CCs) from total VFs present in particular STs or CCs, is shown (Table 8).

**TABLE 3:**
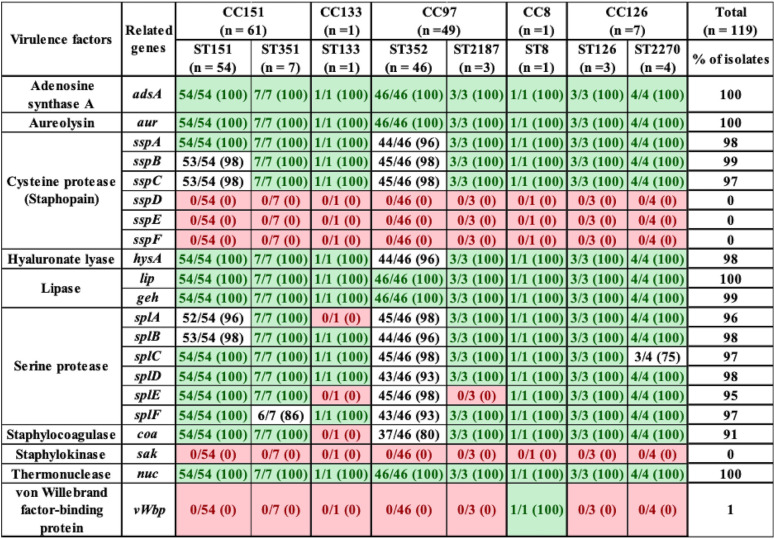
Distribution of exoenzymes in S. aureus isolates grouped according to ST and CC^*a*^

a Light green indicates gene was in all isolates within a given ST; red indicates a gene that was not identified in any isolates within a given ST. Values in parentheses indicate the percentage of isolates that contained a particular VF.

**TABLE 4:**
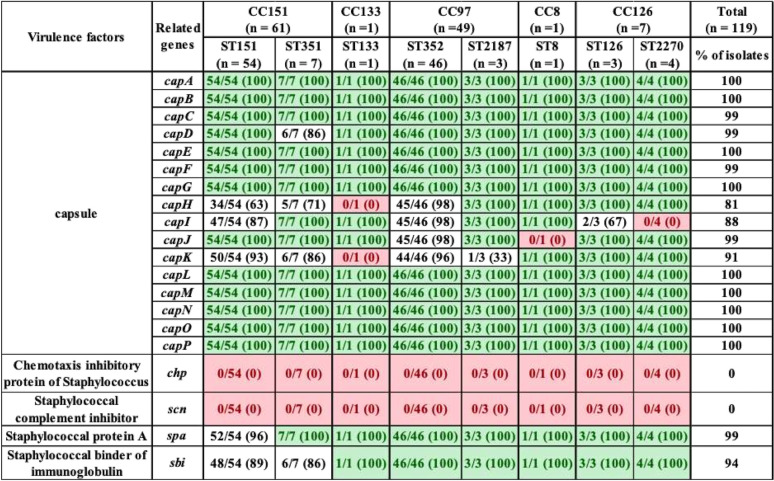
Distribution of host immune evasion genes in S. aureus isolates grouped according to ST and CC^*a*^

a Light green entries indicate gene was in all isolates within a given ST; red entries indicate a gene that was not identified in any isolates within a given ST. Values in parentheses indicate the percentage of isolates that contained a particular VF.

**TABLE 5:**
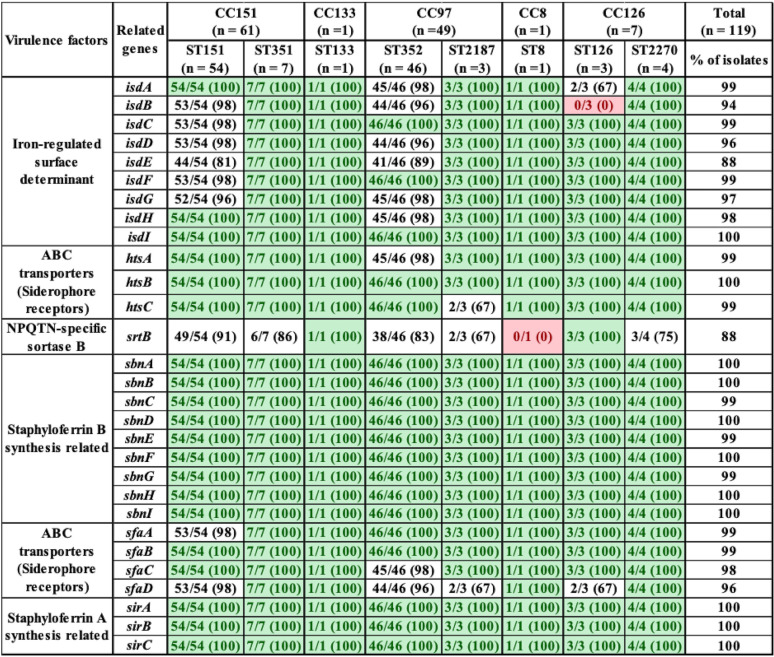
Distribution of iron acquisition- and metabolism-related genes in S. aureus isolates grouped according to ST and CC^*a*^

a Light green entries indicate gene was in all isolates within a given ST; red entries indicate a gene that was not identified in any isolates within a given ST. Values in parentheses indicate the percentage of isolates that contained a particular VF.

**TABLE 6:**
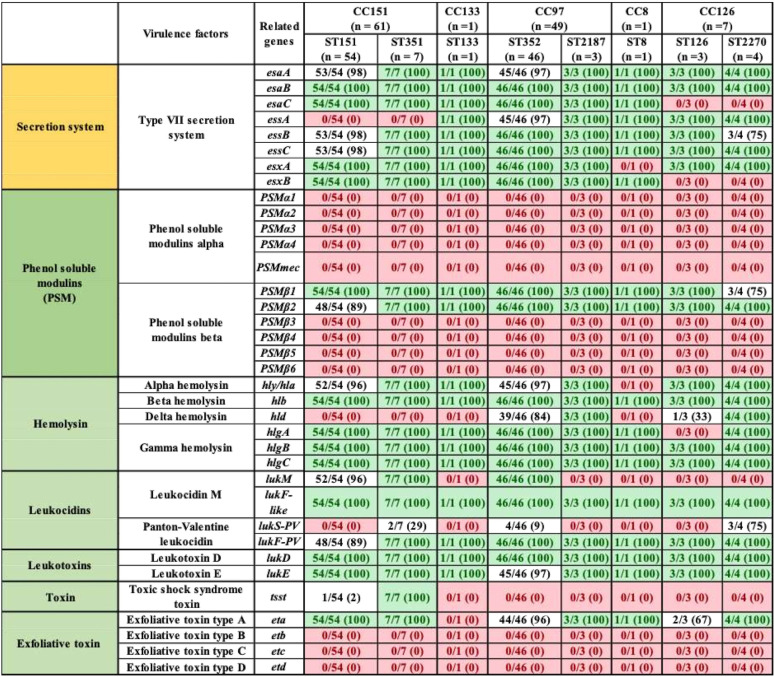
Distribution of different toxin system genes in S. aureus isolates grouped according to ST and CC^*a*^

a Light green entries indicate gene was in all isolates within a given ST; red entries indicate a gene that was not identified in any isolates within a given ST. Values in parentheses indicate the percentage of isolates that contained a particular VF.

**TABLE 7:**
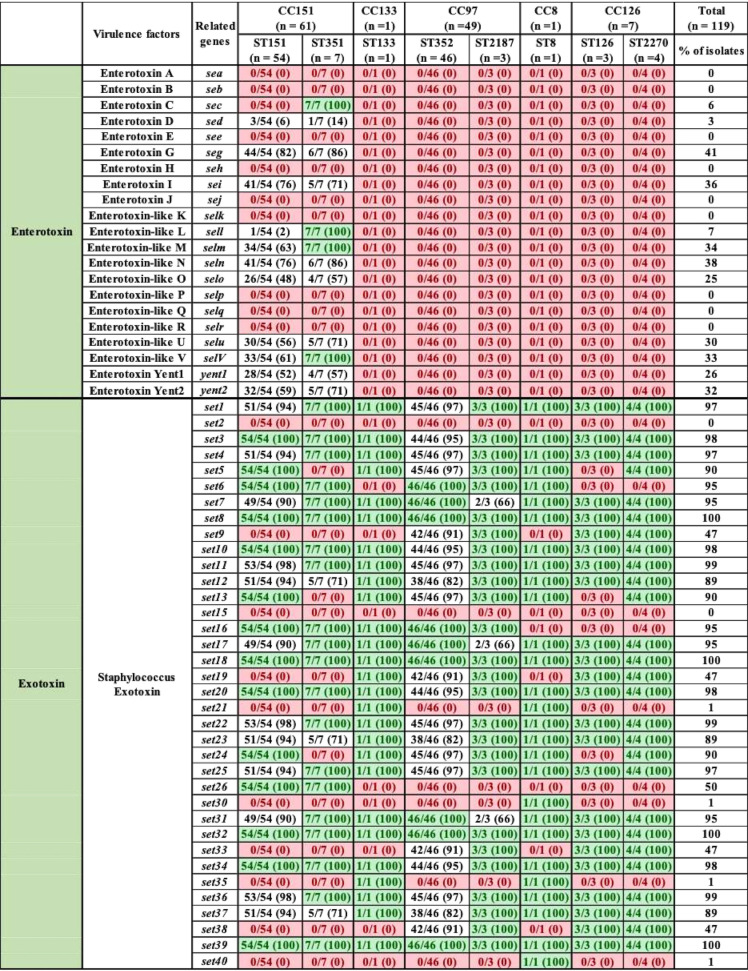
Distribution of different entero- and exotoxins in bovine S. aureus isolates grouped according to ST and CC^*a*^

a Light green entries indicate gene was in all isolates within a given ST; red entries indicate a gene that was not identified in any isolates within a given ST. Values in parentheses indicate the percentage of isolates that contained a particular VF.

**TABLE 8:**
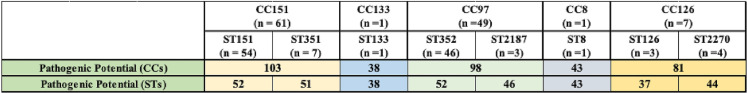
Pathogenic potential of clonal complexes (CC) and sequence types (STs)

**FIG 4:**
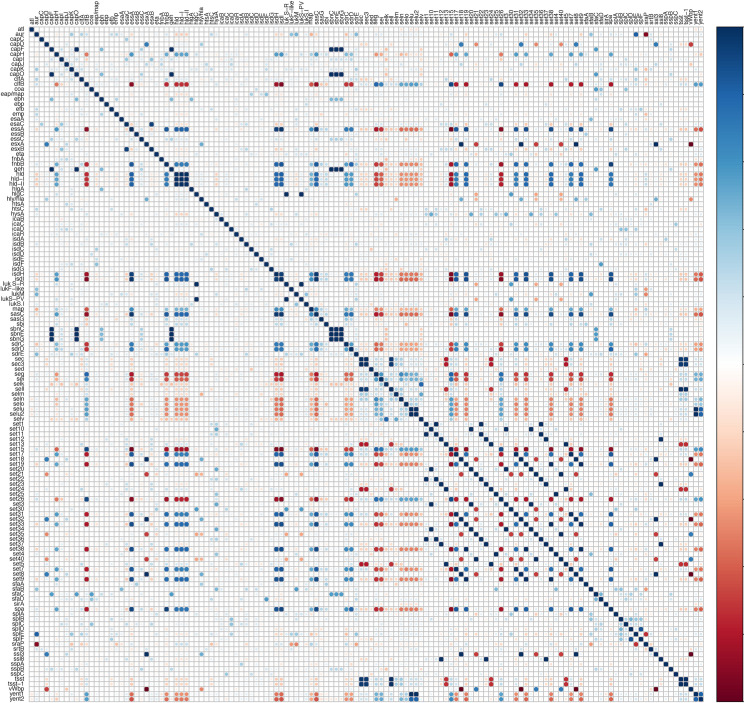
Pairwise associations of virulence factors. Pairwise associations among all virulence genes were detected in 119 bovine Staphylococcus aureus isolates. Associations were computed using phi coefficient. Colors represent type of association (blue, positive; red, negative).

Relationships among and between VFs from 5 categories were investigated using an association plot (Fig. 4). Most associations among VF genes were neutral or very weak. However, there were some strong positive and strong negative associations. For instance, staphyloferrin B synthesis-related genes *sbnC*, *sbnE*, and *sbnG* were strongly positively associated with each other (Fig. 4); these genes also had strong associations with *capF*, *clfB*, *ebh*, *essC*, *geh*, and *isdH.* Similarly, *sdrC* and *sdrD* had positive associations with each other and with *essA*, *fnb*, and *geh* (Fig. 4). Additionally, some interesting patterns of associations were observed among toxin genes. For instance, *seg*, *sei*, *sell*, *selm*, *seln*, *selo*, *selu*, and *selv* were positively associated with each other and with *set26*, *yent1*, and *yent2* but had strong negative associations with *essA*, *fnbB*, *hld*, *sasC*, *sasG*, *srdC*, and *srdD* (Fig. 4). Similarly, many distinctive positive and negative associations were also present among *set* genes. Graphical representation of these and other associations is shown (Fig. 4).

### Phenotypic and genotypic AMR.

Phenotypic resistance was common against beta-lactams (19% of isolates) and sulfonamides (7% of isolates). However, no isolate was resistant to oxacillin or cephalotin. Resistance against pirlimycin, tetracycline, ceftiofur, and erythromycin and to the combination of penicillin and novobiocin was uncommon (3, 3, 3, 2, and 2% of all isolates, respectively). The most common genotypic AMR markers were (i) presence of AMR-associated residues in the dihydropteroate synthase gene deduced amino acid sequence (*folP* gene; all sequenced isolates, ranging from 1 to 11 residues); (ii) chromosomally encoded efflux pump MepA, represented by the *mepA* gene (all isolates), and *tet*(38) (99% of isolates); (iii) multidrug export protein SAV1866 (59% of isolates); and (iv) multidrug efflux pump NorA, represented by the *norA* gene (58% of isolates). No mutations previously described as associated with AMR in S. aureus were detected in the quinolone resistance-determining region of *gyrA*, *gyrB*, *parC*, and *parE* genes. Similarly, no mutations were detected for *rpoB*, *rpoC*, *mprF*, and *cls* genes. Regarding acquired genetic mechanisms, the *blaZ* gene was present in 4 isolates, with 3 resistant to beta-lactams. A single S. aureus isolate phenotypically resistant to erythromycin harbored *ermC*, whereas *tet*(M) and *mecA* were detected in a single isolate resistant to beta-lactams, tetracycline, and sulfonamides. The same isolate contained 11 residues associated with sulfonamide resistance in the deduced amino acid sequence of the *folP* gene.

### Associations between presence of VFs and mastitis.

No association was detected between the total number of VF genes and SCC in original milk samples. Furthermore, the number of VFs of any category was not associated with the severity of inflammatory response or disease severity, as categorized into low, medium, and high SCC or clinical mastitis. Neither STs nor CCs differed in their impact on sample SCC or severity of immune response. Similarly, no clearly identifiable clusters of isolates were detected in *t*-distributed stochastic neighbor embedding (t-SNE) graphs when labeled according to immune response severity.

## DISCUSSION

Several studies have focused on the epidemiology of bovine S. aureus in dairy herds (4, 39–42). In addition, many studies used PCR-based techniques to identify virulence and AMR genes among bovine S. aureus isolates (10, 43–45). However, no large-scale studies have investigated S. aureus virulence and AMR determinants in the context of its genetic diversity, STs, and CCs. Therefore, we conducted WGS of 119 S. aureus isolates and determined STs and CCs. Additionally, we also determined the distribution of 191 VFs and all known ARGs. The MLST analysis clustered 119 S. aureus isolates into 8 distant STs, grouped in 5 CCs. However, the majority of isolates were assigned to CC151 (51%), CC97 (41%), and CC126 (6%). Isolation of these CCs from bovine milk is consistent with most studies (46–49). In many American and European countries, CC151 was the most common and successful clonal type recovered from bovine mastitis outbreaks (46, 47, 49). Similarly, the involvement of CC97 and CC126 in bovine mastitis has been extensively reported from many countries, including South Africa, Brazil, Chile, Italy, Japan, Norway, Spain, The Netherlands, and the United States (46, 49, 50). Isolation of CC97 from humans and other hosts has also been reported (49). However, CC151 and CC126 are confirmed in and are believed to be adapted and limited to cattle (49–52). We also identified 2 other STs (ST8 and ST133), represented by 1 isolate each in our study. ST8 (CC8) is considered a human clone and has mostly been reported in human infections and clinical specimens (49, 53, 54); it is considered the most successful S. aureus lineage, from which a number of major methicillin-resistant S. aureus (MRSA) clones have emerged (50, 55–57). However, CC8 isolation from other hosts, e.g., horses and cows, has also been reported (50), leading to the hypothesis that CC8 has moved from humans to cattle (53, 58), with CC8 MRSA transmitted back to humans (50, 54). We also report the isolation of ST133 (CC133) from bovine milk. The isolation of CC133 from cattle is rare; only a few studies have demonstrated its association with bovine IMI (52, 59). In contrast, the majority of ST133 isolates have been recovered from small ruminants, especially goats and sheep (52, 58, 60). Similar to CC8, the CC133 clone has been proposed to have made the jump from humans to ruminants (58). The most parsimonious explanation regarding the recovery of ST133 from bovine milk is the transfer of ST133 from humans to cattle.

Understanding relationships between strains is important for characterizing pathogen spread. In this study, WGS-based phylogenetic trees were constructed using core proteins and nucleotide sequences. All CGTs and MLSA trees had similar branching of S. aureus isolates. The phylogenic tree constructed from MLSA produced similar larger clades but failed to resolve relationships toward tips of the tree. This was not surprising, as MLSA is based on concatenated sequences of 7 genes, representing only a small fraction of the total genome, and many studies recommend WGS for inferring phylogenies (33, 35, 61). Phylogenetic information obtained from a limited number of genes is influenced by choice of method and selection of evolutionary model of phylogenetic estimations; therefore, it often produces conflicting phylogenies for recent evolutionary descents, represented by tips in phylogenetic trees (34).

The distribution of 191 VFs was determined after grouping these VFs into 5 functional categories. Among 28 adherence genes, 21 were detected in ≥1 isolate. Genes in this category facilitate adhesion and biofilm formation. A hallmark of S. aureus pathogenicity is their capacity to bind to extracellular matrix or to host cells (11, 14, 62). Adhesion is indeed the first step in biofilm formation or invasion of host cells, protecting bacteria from the host immune system and facilitating chronic infection (11, 62). Adhesion relies on the expression of 8 genes (*clfA*, *clfB*, *cna*, *fnbA*, *fnbB*, *srdC*, *srdD*, and *srdE*) that encode a repertoire of surface proteins, called microbial surface components recognizing adhesive matrix molecules (MSCRAMMs) (62), and the subsequent release of biofilm-related proteins (11, 14, 63). The ability of bacterial pathogens to produce biofilms is regarded as a major cause of resistance to antibiotics and was demonstrated to be involved in persistent infections in animals and humans (63–65). Almost all genes of the *ica* operon were detected in S. aureus isolates. The *ica* operon encodes polysaccharide intercellular adhesins (PIA), the earliest recognized and most widely distributed genetic determinant of biofilms (66–68). Interestingly, in this study, an important biofilm-related gene, *aap*, was not detected in any of the S. aureus isolates, whereas another biofilm-associated gene, *bap*, was only detected in 2 isolates. In previous studies, *bap* was described as a cattle-specific pathogenic factor of biofilm formation (11, 63). The presence of all *ica* genes and absence of *aap* and *bap* genes indicates the *ica*-dependent biofilm formation of these isolates, consistent with previous findings (68–70).

Exoenzymes, capsular genes, iron uptake, and type VII secretion system genes were widely distributed among all S. aureus isolates. These genes enable S. aureus to cause infection and survive in bovine udders and make it a successful and devastating bovine pathogen (13, 71, 72). Interestingly, *scn* and the *chp* genes of the immune evasion cluster (IEC), located on β-hemolysin-converting bacteriophages (50, 73) and known to be specific for human isolates (74, 75), were not detected in this study. Among hemolysins, most alpha-, beta-, and gamma-hemolysin genes were identified in all S. aureus isolates, although *hld* was detected only in ST352 and ST126, consistent with other reports (12, 43, 50). The presence of the *hlb* gene was reported to have an antagonistic relationship with IEC genes, as the latter was reported to cause insertional inactivation of *hlb* (50), which was also evident from our results. All STs had *lukED* genes, in agreement with previous studies reporting the presence of *lukED* among bovine isolates (48, 50, 76, 77). However, despite higher prevalence in bovine isolates, *lukED* has also been reported in human isolates (78, 79).

The toxic shock syndrome toxin gene (*tsst*) was detected in ST351 and only 1 isolate of ST151. The importance of toxin secretion by S. aureus in the pathogenesis of mastitis remains unclear. However, superantigens and leukotoxins are considered to have important roles in the initiation and progression of bovine mastitis due to their influence and ability to modulate the immune system (10, 46, 80). The distribution of 12 (of 21) enterotoxin genes in ST151 and ST351, the most prevalent sequence types, was interesting. None of these genes were detected in other STs. Enterotoxins are heat stable and may remain after heat treatment in various dairy products (81–83). Enterotoxins have been associated with staphylococcal food poisoning caused by cow milk or other dairy products (81–83). Interestingly, isolates from the highly prevalent CC97 and CC133 that are strongly associated with CM (52, 58, 84) had no enterotoxin genes. Exotoxin genes were prevalent in S. aureus isolates, and, except for the *set15* gene, all other genes were detected in at least 1 isolate.

Of 191 genes tested, 87 (45%), here defined as core VFs, were detected in all isolates (95% to 100%) of all STs. The presence of these genes in all STs implicate them as having a role in host adaptation and survival in host environments and niches and not to obligately cause mastitis, as not all isolates containing these genes were recovered from clinically diseased animals. However, the presence of these genes may help S. aureus to establish as an opportunistic pathogen. Various VFs (36/191) were not detected in any of our S. aureus isolates. All STs contained 120 to 135 VFs. No association between the number of virulence genes and mastitis was identified; however, the presence of VF genes in genomes does not necessarily relate to gene expression (85) but rather is influenced by multiple factors, e.g., environment, nutritional status, presence of other competing microbes, and host genetics (86–89). The pathogenesis of S. aureus infection is complex and requires systematic participation of multiple VFs to establish disease (11, 13, 86, 90). To understand synergistic or antagonistic links between VF genes, we generated VF association graphs. Further studies focused on unraveling these interactions may extend the understanding of S. aureus pathogenesis.

Development of resistance to antimicrobials can be considered a virulence determinant, as AMR enhances host pathogenesis and allows persistent or chronic infections (91–93). Identifying ARGs is critical to recognize and assess the pathogenic potential of S. aureus. Fewer ARGs were identified in this study than from non-aureus staphylococci (NAS) originating from the same herds (36). This corresponded with reports that S. aureus strains isolated from mastitis cases were less resistant than NAS against commonly used antimicrobials (94, 95). Interestingly, 19% of isolates demonstrated *in vitro* resistance against beta-lactams, whereas *blaZ* or *mec* genes, the two most widespread mechanisms of acquired beta-lactam resistance in S. aureus (96), were detected in roughly 4% of isolates. It remains unclear whether most resistant isolates harbored genetic elements other than ones screened or if bacteria were tolerant to beta-lactams at low concentrations *in vitro*. Of note, beta-lactam-resistant bacteria where no genetic mechanisms of resistance were detected had a range of MICs against penicillin and ampicillin of 0.25 to 8 μg/ml. Therefore, it is unlikely that typical 2-fold variance in MICs would explain nearly 15% of resistant isolates without identified genetic mechanisms of resistance. Here, a single isolate harbored the *mecA* gene, demonstrating that this gene is still uncommon in S. aureus isolated from Canadian dairy herds, in contrast to the higher prevalence observed for S. epidermidis isolated from IMI (36). Similarly, the prevalence of this genetic element in S. aureus isolated from herds in the United States is relatively low compared to the same in NAS (97).

The presence of MDR efflux pumps was observed in all S. aureus isolates. However, to be associated with resistance, these elements would need to be upregulated. Of note, other studies reported the high prevalence of the same elements in *Staphylococcus* spp. (98). Interestingly, the presence of genes encoding MDR efflux pumps, such as NorA and SAV1866, demonstrated an association with several virulence genes, with some virulence genes being more frequent in isolates harboring these MDR efflux pumps, whereas others were uncommon in or absent from the same subset of isolates. In the absence of mechanistic studies, it is unclear whether these patterns reflected a gene interaction that influenced AMR and/or virulence. Nevertheless, WGS facilitates scanning genomes for all known genetic determinants of antibiotic resistance (36, 37), VF genes, and their interactions.

### Conclusions.

Based on WGS, we determined that 119 bovine milk Staphylococcus aureus isolates belonged to 8 sequence types (ST151, ST352, ST351, ST2187, ST2270, ST133, and ST8), 5 clonal complexes (CC151, CC97, CC126, CC133, and CC8), and 18 distinct Spa types. Pan-, core, and accessory genomes of these isolates were composed of 6,340, 1,279, and 2,431 genes, respectively. Phenotypically, resistance was most common against beta-lactams (23 [19%] isolates) and sulfonamides (8 [7%] isolates), whereas resistance was uncommon against pirlimycin, tetracycline, ceftiofur, and erythromycin and to the combination of penicillin and novobiocin (3, 3, 3, 2, and 2% of all isolates, respectively). We also established comprehensive VF gene profiles of 119 S. aureus isolates, calculated the pathogenic potential of CCs and STs, and determined that CC151 (ST151 and ST351) had the highest VF potential, followed by CC97 (ST352 and ST2187) and CC126 (ST126 and ST2270), whereas CC133 (ST133) and CC8 (ST8) had the lowest pathogenic potential. However, the mere presence and number of genes cannot determine the pathogenesis of S. aureus, which is complex and depends on several factors, such as, but not limited to, host health, activation, and expression of virulence genes, host immune system, and geographic influences. Additionally, variations in VF genes among CCs and STs may represent evolution toward adaptation to host or distinct niches or environments within a host. To the best of our knowledge, this was the first study that performed WGS on a large number of S. aureus strains isolated from bovine IMI and determined STs, CCs, and Spa types, computed pan-genomes, determined the distribution of AMR and 191 virulence genes, and calculated the pathogenic potential of distinct STs and CCs.

## MATERIALS AND METHODS

### Isolates.

Isolates were obtained from the National Cohort of Dairy Farms of the Canadian Bovine Mastitis Research Network (99). Briefly, 89 herds from 6 Canadian provinces, selected to be representative of their respective province in terms of bulk tank SCC and housing system, were monitored from February 2007 to December 2008. Milk samples were collected according to 3 sampling schemes: (i) all samples from clinical mastitis cases; (ii) weekly or biweekly samples of 15 randomly selected lactating cows from each herd; and (iii) milk samples prior to drying off and after calving. Overall, 115,294 milk samples were obtained from 5,157 lactating cows. Staphylococcus aureus was detected in 3,387 milk samples obtained from 1,042 cows in all 89 herds (range, 1 to 27 isolates per cow). From this total, a random selection of 119 isolates from 119 cows was done. Details about the number of isolates and unique herds of origin were grouped by SCC level for nonclinical samples, and a separate category for clinical mastitis samples, are given in Table 1.

### DNA extraction and whole-genome sequencing.

Isolates were grown on 5% sheep blood agar plates (BD Diagnostics, Mississauga, ON, Canada) at 37°C for 24 h to yield single colonies, suspended in Bacto brain heart infusion broth (BD Diagnostics), and incubated at 37°C overnight. Genomic DNA was extracted with a DNeasy blood and tissue kit (Qiagen, Toronto, ON, Canada) by following the protocol for Gram-positive bacteria. The concentration and quality of genomic DNA were determined with a NanoVue Plus spectrophotometer (GE Healthcare Life Sciences, Mississauga, ON, Canada) and the Qubit 2.0 fluorometer (Invitrogen, Burlington, ON, Canada). Each DNA sample was diluted to a final concentration of 0.2 ng/μl. Paired-end DNA libraries of 250 bp were prepared using a Nextera XT DNA library preparation kit (Illumina, San Diego, CA, USA) and samples sequenced with an Illumina MiSeq platform (Illumina). Primary data analysis for quality control was done on the MiSeq platform.

### Genome assembly and annotation.

Genome assemblies and annotations were done using an in-house pipeline, as described previously (34, 100). Briefly, poorly sequenced regions and Illumina adapter sequences from sequence reads obtained from the MiSeq platform were identified and removed using cutadapt (101), implemented in Trim Galore! 0.4.0 (with default parameters). Filtered reads were assembled into contigs using the *de novo* assembly program SPAdes version 3.6.0 (102), employing built-in error correction and default parameters. Sequencing depth of coverage for each genome was determined by mapping reads back to the assembled genome using BWA 0.7.12-r1039 (103). The identification of coding sequences (CDS) and genome annotations was performed with Prokka 1.12 (104), using the provided (with Prokka) *Staphylococcus* database. Briefly, protein-coding genes, tRNAs, and rRNAs were predicted by Prodigal v2.6.2 (105), Aragorn v1.2.36 (106), and RNAmmer v1.2 (107), respectively. The quality of assembled genomes and assembly metrics was determined using Quast (108). The entire genome assembly process was automated using the Snakemake workflow engine (109).

### Determination of STs and spa types.

Multilocus sequence typing (MLST) was performed to determine distinct sequence types (STs) (110). The S. aureus MLST scheme is based on 7 housekeeping genes, *arcC* (carbamate kinase), *aroE* (shikimate dehydrogenase), *glpF* (glycerol kinase), *gmk* (guanylate kinase), *pta* (phosphate acetyltransferase), *tpi* (triosephosphate isomerase), and *yqi* (acetyl coenzyme A acetyltransferase) (111). Full-length sequences of these 7 genes from 119 S. aureus genomes were obtained using BLAST+ 2.5.0 (112) and compared at each locus with those of the known alleles in the S. aureus MLST database (https://pubmlst.org/saureus) to obtain allelic profiles and to determine STs. Clustering of STs into complexes (CCs) was done using eBURSTv3 (113) based on predictor founders. Spa types (*t*) were predicted using spaTyper v1.0 webserver (114) from the Center of Genomic Epidemiology (https://cge.cbs.dtu.dk/services/spatyper). The spa typing technique compares the 21 to 27 polymorphic VNTR in the 3′ coding region of staphylococcal protein A (spa) to assign a unique repeat code corresponding to its spa type.

### Phylogenetic analyses.

**(i) Core genome phylogenies according to protein trees.** To understand evolutionary relationships and determine diversity among S. aureus isolates, phylogenetic trees of these isolates were constructed using both a core set of proteins and a data set of 400 ubiquitous marker proteins (115). The core protein tree (CPT) was constructed as described previously (34). Briefly, the core set of S. aureus protein families (80% sequence identity and 80% sequence length), present in ≥90% of input genomes, was identified using the CD-HIT program (116). Protein families that contained potential paralogous sequences (duplicated sequence in the same genome) were excluded from further analysis. Multiple-sequence alignments (MSA) of each protein family were performed using the Clustal Omega (117) algorithm. Aligned amino acid positions with gaps in >50% of genomes were excluded from further analysis. The remaining amino acid positions were concatenated to create a combined data set. Poorly aligned regions from this concatenated alignment were removed using Gblocks 0.92 (118). This combined data set was further trimmed with trimAl (119). Maximum-likelihood (ML) trees based on this alignment were constructed using FastTree 2.1 (120), using the Whelan and Goldman substitution model (121), and a phylogenetic tree based on 400 marker proteins was constructed using PhyloPhlAn (115).

**(ii) Identification of SNP sites and construction of SNP trees.** For SNP identification, a whole-genome alignment-based method, kSNP v3.021 (122), and an alignment-free sequence analysis method, Parsnp v1.2 (123), were used to identify and construct SNP trees. Prior to kSNP3 analysis, the kchooser script was used to determine optimum k-mer size (122). The input file for kSNP3 analysis was created using the *MakeKSNP3infile* program in fully automatic mode. The kSNP3 analysis was done with the following parameters: -k 19 -ML -NJ -core -vcf -CPU 30. For Parsnp analysis, default parameters and autorecruitment of the reference genome were followed to construct core whole-genome SNP alignment. Within the core genome SNP alignment, aligned columns with recombination signals were detected and removed by PhiPack (124). An ML tree, based on the final alignment of core genome SNPs, was constructed using FastTree 2.1 (120). The placement of SNPs over the phylogenetic tree was visualized using the ginger program (123).

### Multilocus sequence analysis.

Multilocus sequence analysis was performed on nucleotide sequences of 7 housekeeping genes (*arcC*, *aroE*, *glpF*, *gmk*, *pta*, *tpi*, and *yqi*). Full-length sequences of these genes (from 119 S. aureus isolates) were obtained using BLAST+ 2.5.0 (112). MSAs for each of these genes were created using MUSCLE v3.8.31 (125). Individual alignments were concatenated to create a combined data set. Poorly aligned regions from this concatenated alignment were removed using Gblocks 0.92 (118). An ML tree based on 100 bootstrap replicates was constructed using MEGA 6.0 (126), using the general time-reversible model (127).

### Pan-genome analysis.

The pan-genome of 119 S. aureus isolates was computed with Roary v 3.12.0 (128). In Roary analysis, GFF files of all S. aureus isolates produced by Prokka (104) were used as input files for Roary, which uses the CD-HIT algorithm (116) to cluster orthologous gene families. Multiple-sequence alignment of gene families was performed using the PRANK v 0.170427 program (129). For Roary analysis, genes present in ≥99% of input genomes and sharing ≥90% sequence identity were considered core. The pan-genome was represented as the core genome (shared by >99% of strains), accessory genome (genes present in >2 strains but not in all), and unique genome (genes unique to individual strains). The total pan-genome was also shown as core (99% ≤ strains ≤ 100%), soft core (95% ≤ strains < 99%), shell (15% ≤ strains < 95%), and cloud (0% ≤ strains < 15%). Functional annotations of core, accessory, and unique genes were obtained after comparing these sequences with COG and KEGG databases implemented in BPGA v1.3 (130). To visually observe distributions of the pan-genome to S. aureus isolates, a gene_presence_absence table obtained from Roary analysis was superimposed onto CGT using the roary_plots script (128). Distributions of the pan-genome to individual isolates were plotted with the Roary2SVG script (128).

### Collection of virulence and AMR genes and classification of VFs.

For VFs, a comprehensive VF data set of staphylococci (CVFS) created in our previous study (38) was used. Briefly, CVFS was developed by collecting S. aureus VF sequences from the VFDB database (131), Victors database (http://www.phidias.us/victors/), PATRIC database (132), and phenol-soluble modulin sequences from the UniProtKB database (133). Virulence factors (*n* = 191) were classified into 5 functional categories: adherence (*n* = 28), exoenzymes (*n* = 21), host immune evasion (*n* = 20), iron uptake and metabolism (*n* = 29), and toxins (*n* = 93). The 28 VFs of the adherence category were accumulation-associated protein (*aap*), biofilm-associated surface protein Bap (*bap*), autolysin (*atl*), clumping factors (*clfA* and *clfB*), collagen adhesion (*cna*), elastin binding protein (*ebp*), fibronectin binding proteins (*ebh*, *efb*, *uafA*, *fnbA*, and *fnbB*), extracellular adherence/major histocompatibility complex analogous protein (*eap-map*), cell wall surface anchor family proteins (s*asC*, *sasG*, and *sasP*), intercellular adhesins (*icaA*, *icaB*, *icaC*, *icaD*, and *icaR*), and Ser-Asp rich fibrinogen-binding proteins (*sdrC*, *sdrD*, *sdrE*, *sdrF*, *sdrG*, *sdrH*, and *sdrI*). Exoenzymes consisted of adenosine synthase A (*adsA*), aureolysin (*aur*), cysteine proteases (*sspA*, *sspB*, *sspC*, *sspD*, *sspE*, and *sspF*), hyaluronate lyase (*hysA*), lipases (*lip* and *geh*), serine proteases (*splA*, *splB*, *splC*, *splD*, *splE*, and *splF*), staphylocoagulase (*coa*), staphylokinase (*sak*), thermonuclease (*nuc*), and von-Willebrand factor-binding protein (*vWbp*).

The host immune evasion category consisted of capsular genes (*capA*, *capB*, *capC*, *capD*, *capE*, *capF*, *capG*, *capH*, *capI*, *capJ*, *capK*, *capL*, *capM*, *capN*, *capO*, and *capP*), chemotaxis inhibitory protein (*chp*), staphylococcal complement inhibitor (*scn*), staphylococcal protein A (*spa*), and staphylococcal binder of immunoglobulin (*sbi*) gene. The iron uptake and metabolism category included 9 iron-regulated surface determinants (*isdA*, *isdB*, *isdC*, *isdD*, *isdE*, *isdF*, *isdG*, *isdH*, and *isdI*), 7 ABC transporters (also known as siderophore receptors; *htsA*, *htsB*, *htsC*, *sfaA*, *sfaB*, *sfaC*, and *sfaD*), 12 staphyloferrin A and B synthesis-related genes (*sirA*, *sirB*, *sirC*, *sbnA*, *sbnB*, *sbnC*, *sbnD*, *sbnE*, *sbnF*, *sbnG*, *sbnH*, and *sbnI*), and 1 sortase B (*srtB*). Toxin genes included genes for alpha-, beta-, delta-, and gamma-hemolysins (*hly-hla*, *hlb*, *hld*, *hlgA*, *hlgB*, and *hlgC*), 4 genes for leukocidins, including leukocidin M (*lukM* and *lukF-like*) and Panton-Valentine leukocidins (*lukS-PV* and *lukF-PV*), 2 leukotoxins (*lukD* and *lukE*), toxic shock syndrome toxin (*tsst*), 4 exfoliative toxins (*eta*, *etb*, *etc*, and *etd*), 8 genes of type VII secretion system (*esaA*, *esaB*, *esaC*, *essA*, *essB*, *essC*, *esxA*, and *esxB*), and 11 genes for phenol-soluble modulins, including the 5 alpha (*PSMα1*, *PSMα2*, *PSMα3*, *PSMα4*, and *PSMmec*) and 6 beta (*PSMβ1*, *PSMβ2*, *PSMβ3*, *PSMβ4*, *PSMβ5* and *PSMβ6*) genes plus 21 enterotoxin and 36 staphylococcal exotoxin (*set*) genes.

For AMR gene (ARG) screening, a data set of ARGs was constructed, as described previously (36), after combining AMR gene sequences from 4 databases: (i) ARG-ANNOT v3 (Antibiotic Resistance Gene-ANNOTation), (ii) MegaRES v1.0.1 (134), (iii) Comprehensive Antibiotic Resistance Database v1.1.6 (CARD) (135), and (iv) ResFinder from the Center for Genomic Epidemiology (136).

### Identification of virulence and antimicrobial resistance genes.

The presence of VFs and ARGs was determined as described previously (36, 38). For this purpose, a local blastdbs of 119 S. aureus was created with the makeblastdb application from BLAST+ 2.5.0 (112). BLASTp searches of CVFS and ARG sequences were done against S. aureus genomes. Homology between query protein sequences and blast hits was determined by calculating *H* scores (36, 137). The *H* scores between protein sequences, labeled *Ha* (where *a* represents amino acid), were calculated using the formula *Ha* = *Qid* × *Lm/Lq* (36), with *Qid* representing the level of BLASTp identities between query sequence and identified protein sequence (range, 0 to 1), *Lm* representing length of the matching sequence from the hit, and *Lq* denoting the length of the query sequence. Cutoffs of 80% sequence similarity and 70% query length coverage were used for initial searches. All genomic hits that met the minimal cutoff for each individual query were selected at this stage. A final blast hit table containing all possible hits for all query sequences from all S. aureus genomes was imported into R v.3.4.2 (138). Hits from each query sequence were then arranged according to *Ha* score, using dplyr version 0.7.2 in R (139). From this list, only hits with highest *Ha* score (highest sequence similarity and query length coverage) were selected as potential VFs and ARGs in S. aureus genomes, whereas the remainder were discarded. After the identification of putative S. aureus VFs, to confirm orthology between identified putative S. aureus VF and CVFS sequences, reciprocal blast searches between the putative S. aureus VFs and CVFS database were done (140). Putative S. aureus VF sequences that failed to match corresponding VFs from the CVFS database as best hits in reciprocal blast searches were not considered true orthologs and were excluded from further analysis.

For AMR genes, protein sequences of all top hits were used as queries against the nonredundant (nr) database from NCBI using BLASTp (112). The best hit was considered definitive as long as it had >80% coverage and percent identity with the query (36). For AMR genes that required additional confirmation (substitutions and residues composition), pairwise alignments of putative AMR genes with those of reference genes (36) were done using MEGA 6.0 (126). The ARGs obtained from 119 S. aureus isolates were screened for the presence of residues known to be associated with AMR in S. aureus.

### Antimicrobial resistance profiles.

For all isolates, the phenotypic AMR profile was determined using the MIC, in accordance with Clinical Laboratory and Standards Institute (CLSI) guidelines (141). Antimicrobials and concentrations (in micrograms per milliliter) evaluated were the following: ampicillin (0.128), ceftiofur (0.5 to 4), cephalothin (2 to 16), erythromycin (0.25 to 4), oxacillin plus 2% NaCl (2 to 4), penicillin (0.06 to 8), penicillin-novobiocin (1-2 to 8-16), pirlimycin (0.5 to 4), tetracycline (2 to 16), and sulfadimethozine (32 to 256). Breakpoints were defined according to CLSI criteria for animals (141, 142). Isolates were classified as either susceptible or resistant, whereas isolates with MIC equal to intermediate breakpoints were considered resistant. Antimicrobial-free wells were included in all plates, and S. aureus ATCC 29213 was used as a quality control strain for all tests.

### Associations between presence of VFs and mastitis.

Associations between the presence of VFs with mastitis were assessed by conducting statistical analyses using the ordinal package (143) and base functions in R v.3.4.2 (138), with a *P *value of <0.05 considered significant. Clustering and dimensionality reduction analyses were conducted using Python and the package Scikit-Learn (144). Relationships between measures of mastitis and VFs were examined after dichotomizing genes (presence or absence) in all isolates. For samples collected from animals without clinical symptoms of mastitis, the association of the natural logarithm of SCC (LnSCC) with the number of VFs present was assessed using linear regression; the outcome was the LnSCC, and predictor was the total number of VFs or number of virulence genes of a given type in the isolate. Model fit was assessed using multiple *R*^2^, whereas the strength of association was assessed by examining the coefficient for the predictor variable. To include samples from clinical mastitis, there were 4 outcome categories based on SCC and sample type, low SCC (≤150,000 cells/ml), medium SCC (150,000 < SCC ≤ 250,000 cells/ml), high SCC (>250,000 cells/ml), and clinical mastitis (isolated from a quarter with clinical mastitis) and rank. To assess associations between inflammatory response and disease severity and virulence genes, ordinal logistic regression was conducted using the outcome defined above and the total number of virulence genes or number of virulence genes of a given category in the isolate as a predictor. Coefficients estimated using this regression represented the likelihood of a more severe mammary response (measured as SCC) for each additional virulence factor identified. For dimensionality reduction and to visualize distributions of VFs within isolates, *t*-distributed stochastic neighbor embedding (t-SNE [145]) was conducted using the manifold.t-SNE module within Scikit-Learn (144). Genetic distributions were reduced to 2 or 3 dimensions and visually examined. Plots were labeled with severity of immune response and geographical region (Canadian province) from which samples were isolated to identify potential clusters of interest.

### Associations between STs, CCs, and mastitis.

Similar to models described above, clinical mastitis isolates were excluded, after which LnSCC was modeled, or mammary inflammation severity was categorized into 4 ordinal categories. Linear regression models were used to assess differences in mean LnSCC between STs and CCs, whereas ordinal logistic regression models were used to determine association between ST or CC and severity of mammary inflammation.

### Associations between the presence of virulence genes.

Using the same dichotomized values for the presence of virulence genes, associations with STs and clonal complexes were also assessed. Both CCs and STs were treated as a categorical outcome variable, and a multinomial logistic regression using the package nnet (146) was conducted, with predictors being the total number of VFs or number of VFs of a given category in an isolate. Regression coefficients from a multinomial logistic regression model were interpreted as how much more or less likely an isolate with a given number of virulence genes was to belong to a specific ST or CC compared to the baseline. Regional distributions of both STs and CCs were also assessed using a multinomial logistic regression with STs or CCs as the outcome and region as the only predictor. To visualize genetic distributions, the same components derived during the t-SNE described above were plotted and labeled with either STs or CCs to identify potential clusters.

### Data availability.

All whole-genome sequencing data used in this study are available without restriction from NCBI under BioProject no. PRJNA599195.
